# Role of blood flow in endothelial functionality: a review

**DOI:** 10.3389/fcell.2023.1259280

**Published:** 2023-10-13

**Authors:** Hui Lin Zhou, Xi Zhuo Jiang, Yiannis Ventikos

**Affiliations:** ^1^ School of Mechanical Engineering and Automation, Northeastern University, Shenyang, China; ^2^ Department of Mechanical Engineering, Monash University, Melbourne, VIC, Australia

**Keywords:** flow shear stress, endothelial cells, mechanotransduction, endothelial functionality, endothelial glycocalyx

## Abstract

Endothelial cells, located on the surface of blood vessel walls, are constantly stimulated by mechanical forces from the blood flow. The mechanical forces, i.e., fluid shear stress, induced by the blood flow play a pivotal role in controlling multiple physiological processes at the endothelium and in regulating various pathways that maintain homeostasis and vascular function. In this review, research looking at different blood fluid patterns and fluid shear stress in the circulation system is summarized, together with the interactions between the blood flow and the endothelial cells. This review also highlights the flow profile as a response to the configurational changes of the endothelial glycocalyx, which is less revisited in previous reviews. The role of endothelial glycocalyx in maintaining endothelium health and the strategies for the restoration of damaged endothelial glycocalyx are discussed from the perspective of the fluid shear stress. This review provides a new perspective regarding our understanding of the role that blood flow plays in regulating endothelial functionality.

## 1 Introduction

The vascular system is one of the earliest recorded systems in human history (Slowey and Nyhan, 2022). Fluid shear stress induced by the blood flow plays a key part in maintaining homeostasis and is intimately related to vascular pathology (Traub and Berk, 1998; Tun et al., 2019). Endothelial cells, the cellular monolayer lining the inner surface of blood vessels, are in direct contact with the flowing blood and their response to hemodynamic forces directly affects the health of blood vessels (Baratchi et al., 2017). The intensive interactions between endothelial cells and fluid shear stress, therefore, are pivotal for controlling inflammatory responses, modulating the expression of adhesion molecules, cytokines and chemokines, and regulating the formation and destruction of thrombi (Chiu and Chien, 2011; Davis et al., 2015). The endothelial glycocalyx (EG), a layer of gel-like glycoproteins on the apical surface of endothelial cells, participates in maintaining the homeostasis of organs and blood vessels by fulfilling a variety of functions, including mechanotransduction, and by varying its configuration. The EG might be structurally and functionally different in distinct organs, thus revealing an unexpected level of structural complexity. Typically, an EG unit is composed of glycosaminoglycan (GAG) chains and core proteins which anchor the GAGs to the cell membrane (Jiang et al., 2021b). The GAGs in the EG include hyaluronic acid, heparan sulfate, chondroitin sulfate and sialic acid. The GAG chains feature a high density of negative charges and the electrostatic interactions between the GAG chains and some charged blood components allow the GAG chains to act as a microvascular barrier for the transport of ions (Jiang et al., 2019b; Jiang et al., 2020), macromolecules (Shi et al., 2021) and cells (Jiang et al., 2018c; Nishida et al., 2023). For example, the electrostatic repulsion causes albumin to move away from the vascular wall and towards the center of the vascular lumen (Suzuki et al., 2022). A previous review has summarized the mechanisms of endothelial permeability for transcellular and paracellular transport from the perspectives of molecular affinity, size and charge characteristics (Komarova and Malik, 2010).

Endothelial cells are exposed to flow of various patterns and hemodynamic forces throughout the vascular system (Chiu and Chien, 2011). As the “antennas” of endothelial cells, EG is an active mechano-sensor to transduce mechanical signals from the blood flow and transmit the signals to the endothelial cells. Such a process is often termed mechanotransduction. The mechanical forces from the blood flow drive the movements of endothelial cells, and the alignment of endothelial cells varies with the blood flow patterns. For example, porcine aortic valve endothelial cells elongate and align parallelly to the flow direction under continuous perfusion with a shear stress of 0.47 dyne/cm^2^, while they are perpendicular to the flow direction when porcine aortic valve endothelial cells are under stable or aortic pulsation blood flow with a shear stress of 8.9 dyne/cm^2^ (Lee et al., 2018). Indeed, from the viewpoint of fluid mechanists, the endothelial cell behavior in flow is a quintessential fluid-structure-interaction problem: the flow modifies the alignment of endothelial cell as well as the configuration of EG, and EG, in turn, changes the flow field and thereby the fluid shear stress (Jiang et al., 2018a; Jiang et al., 2018b; Jiang and Ventikos, 2022). Mechanotransduction is, therefore, an interactive process.

The normal expression of endothelial cell genes is essential for the maintenance of vascular homeostasis and integrity, whilst the malfunction of endothelial cells could lead to infectious diseases (such as sepsis), kidney disease, cardiovascular diseases (such as atherosclerosis, diabetes and hypertension) (Kalagara et al., 2018; Machin et al., 2019; Dogné and Flamion, 2020; Xu, 2020; Weinbaum et al., 2021; Mugerli et al., 2022). As endothelial cells are inherently exposed the blood and fluid shear stress, clarifying their sensing mechanisms for shear stress and the relationship between blood flow and homeostasis of the internal environment contributes to the understandings of the physiology and pathology of the vascular system.

In the past decades, researchers have investigated the relationship among endothelial cells, fluid shear stress and vascular physiology and pathology from various aspects, and review papers highlighting the up-to-date research progress have been published. Table 1 summarizes the recent review papers and labels their research of interest. The foci of these review papers are spotlighted by four factors relating to the functionality of endothelial cells, i.e., blood flow pattern, mechanotransduction, flow field modified by EG and vascular pathology if endothelial cells are malfunctioning. Here, the conventional definition of the mechanotransduction is used, i.e., mechanotransduction only refers to the process that EG responds to the flow.

**TABLE 1:** Recent reviews focusing on flow and endothelial cell functionality.

Year	Blood flow pattern	Mechanotransduction	Flow field changed by EG	Vascular pathology	Ref.
2017	●	●	—	—	Baratchi et al. (2017)
2019	—	●	—	●	Machin et al. (2019)
2020	●	●	—	●	Dominic et al. (2020)
2020	—	●	●	●	Souilhol et al. (2020)
2020	—	●	—	●	Goligorsky and Sun (2020)
2020	—	●	—	●	Dogné and Flamion (2020)
2020	●	●	—	●	Xu (2020)
2021	—	●	—	●	Ashby and Mack (2021)
2021	—	●	—	—	Jiang et al. (2021b)
2022	●	●	—	—	Meng et al. (2022)
2022	—	●	●	—	Goligorsky (2022)
2022	—	●	—	●	Suzuki et al. (2022)
2022	—	●	—	●	Cusack et al. (2022)
2023	—	●	—	●	Cheng et al. (2023)

According to Table 1, most review papers focus on the mechanotransduction of endothelial cells and the pathologies of EG-related diseases, whilst only a limited number of papers talk about blood flow patterns and the modified flow field as a response to EG configurational changes. In this review, we will summarize the latest findings about the effects of blood flow patterns on the physiological function expression of endothelial cells, and special focus will be given to the modified flow field by EG configurational changes. The vascular pathology due to the malfunction of EG is also reviewed. This paper will contribute to a comprehensive summarizing, and we hope understanding, of the role of flow in regulating endothelial functionality.

## 2 Flow patterns, fluid shear stress and endothelial cells

Blood flow can be laminar, transitional or turbulent, depending on the blood flow rate, the properties of blood, the blood vessel diameter, and other local geometric characteristics of the vessel. The characteristics of flow are determined by a dimensionless number called the Reynolds number (*Re*), which is calculated using Eq 1:

$$Re=\rho \bar{U}D∕\mu ,$$ (1)

where *ρ* is the density of the blood, $\bar{U}$ is the average velocity of the blood flowing through the blood vessels, *D* is the diameter of the blood vessels, and *μ* is the viscosity of the blood. For a uniform, cylindrically shaped straight vessel, the flow maintains its laminar flow characteristics at *Re*<1000; the flow experiences a transition from laminar to turbulent regime at 1000<*Re*<2000, and becomes turbulent at *Re*>2000 (Ku, 1997). It is noteworthy that the exact numbers where these transitions happen are not so important and may vary depending on the situations. For example, the critical Reynolds number for such a transition is 2300, as originally described by Reynolds, (1883). Previous studies have demonstrated that the blood flow in most of the circulatory system is laminar, whilst in a few regions, such as artery branch points, severely stenotic arteries, stenotic heart valves at peak systole and aneurysms, the flow is disturbed (Baratchi et al., 2017).

Hemodynamic stimulation, including the fluid shear stress driven by pulsatile blood flow and the solid circumferential stress driven by pulsatile blood pressure and associated strain, acts simultaneously on endothelial cells and vascular smooth muscle cells, modulating numerous biological activities (Amaya et al., 2015). The fluid shear stress is a frictional force from the blood flow, parallel to the blood vessel walls, and exerted on the endothelium (Chien, 2007; Tarbell et al., 2014). The fluid shear stress is the product of the blood viscosity (*μ*) and the spatial gradient of blood velocity at the vascular wall (d*U*/dy), as shown in Eq 2 in simple unidirectional flow:

F=μ×dU∕dy, (2)

where *F* represents the fluid shear stress which is expressed in the unit of force per unit area (i.e., N/m^2^, Pascal, or dyne/cm^2^, with 1 N/m^2^ = 1 Pa = 10 dyne/cm^2^) (Chatzizisis et al., 2007). The shear stresses on endothelial cells have a wide range over the vasculature: the arterial shear stresses range from approximately 10 dyne/cm^2^ in the aorta to 50 dyne/cm^2^ in smaller arteries; the venous circulating fluid shear stress is relatively low, ranging from 1 to 20 dyne/cm^2^ (Givens and Tzima, 2016). Based on the flow pattern, fluid shear stress can be further categorized as laminar shear stress, oscillatory shear stress and pulsatile shear stress. Laminar shear stress is highly ordered and streamlined, which maintains the vascular homeostasis by activating mechanoreceptors, aligning endothelial cells along the longitudinal axis of blood vessels, and regulating the expression of intracellular signaling pathways, specific genes and proteins (Tresoldi et al., 2015). Oscillatory shear stress originates from the disturbed blood flow in irregular arterial regions, inducing pro-atherogenic phenotypes in endothelial cells and induces the development of plaques. *In vitro* experiments have confirmed that oscillatory shear stress can participate in cellular endothelial response, triggering hemostatic reactions and vascular remodeling (Storch et al., 2018). Periodic acceleration generated by motion can generate pulsatile shear stress on the vascular endothelium, and pulsatile shear stress could activate the production of a large number of cardiac and cellular protective mediators, such as endothelial nitric oxide, prostacyclin and tissue plasminogen activator (Martínez et al., 2008).

Abnormal shear stress generated by multidirectional or disturbed blood flow will lead to vascular dysfunction, inflammation and injury, thus inducing pathological processes such as atherosclerosis, thrombosis and arterial hypertension (Kwak et al., 2014; Min and Schwartz, 2019). When endothelium is cultured in wells swirled on an orbital shaker, multidirectional shear stresses act on ECs. The areas where multidirectional shear stresses are applied typically have a particularly high prevalence of lesions (Mohamied et al., 2015). At the center of the swirling well, low magnitude multidirectional shear stress induced by the swirling medium dominates; high magnitude and unidirectional flow is generated at the edge (Warboys et al., 2019). Previous studies suggest ECs cultured at the center of the well were influenced by the atherogenic flow and more pro-inflammatory and fewer homeostatic genes were expressed. Higher permeability, apoptosis and senescence together with more frequent endothelial-to-mesenchymal transition were observed on ECs at the center of the well compared to those at the edge (Ghim et al., 2021). The disturbed flow could trigger an increase in endothelial cell proliferation, which is an early symptom of site-specific atherogenesis (Joshi et al., 2020). Conversely, physiologically high laminar shear stresses are atheroprotective (Souilhol et al., 2020; Zeng et al., 2021). For example, *in vitro* studies verified that when the laminar shear stress is greater than or equal to 12 dyne/cm^2^ without oscillation, the protein expression level and activity of eNOS (the basic molecule for the synthesis and releases of the helpful vasodilator, antioxidant, and anti-inflammatory mediator—nitric oxide) in cultured endothelial cells and intact blood vessels are upregulated (Jin et al., 2003; Boo et al., 2006). Another study reported an increase in tumor suppressor gene in the bovine aortic endothelial cells after being exposed to laminar shear stresses of 3 dyne/cm^2^ or higher for 24 h, but when the shear stress was tuned down to 1.5 dyne/cm^2^ the gene level did not increase (Lin et al., 2000). Sustained laminar shear stress, together with the sparing endothelial cells from apoptosis, is capable of preventing the endothelial cells apoptosis induced by cytokines, oxidative stress, serum starvation, or cytoskeletal disruption. Pulsating and high laminar shear stress can exert adverse effects on the blood-brain barrier. As reported in an *in vitro* experiment (Garcia-Polite et al., 2017), the permeability of the brain endothelial monolayer increased and the expression of tight junction proteins decreased under pulsating flow and high laminar shear stress.

Flow adaptations can affect endothelial gene expression, and cells grown under flow conditions respond differently to changes in shear stress compared with those that are grown under static conditions (Raaz et al., 2014). For instance, unidirectional flow can affect the morphology of human umbilical vein endothelial cells more significantly than bidirectional flow or static culture. In a previous study, human umbilical vein endothelial cells were cultured in a static medium for 13 days and then subjected to stable shear stress stimulation at a rate of 12 dyne/cm^2^ for 24 h. After 24 h of shear exposure, human umbilical vein endothelial cells were elongated and their long axis aligned with the flow direction. However, after 24 h of recovery in the static medium, human umbilical vein endothelial cells returned to their original forms (Bai and Wang, 2014). Another study (Inglebert et al., 2020) reported that after long-term exposure to physiological shear stress, a reduction in fluid shear stress could cause endothelial cells to undergo actin cytoskeleton reorganization and phenotypic changes. Indeed, an increasing number of papers have evidenced the different behavior of endothelial cells under varying fluid shear stress stimulations. The recent papers reporting the roles of fluid shear stress in modifying endothelial cell behavior were summarised in Table 2.

**TABLE 2:** Recent articles highlighting the function of fluid shear stress in modifying endothelial cell behavior.

Year	Key findings	Ref.
2023	Laminar shear stress maintains vascular homeostasis which relies on specific mechanosensors on endothelial cells and the corresponding signal transduction pathways, while oscillatory shear stress could promote the pro-inflammatory phenotype of endothelial cells.	Cheng et al. (2023)
2022	Pulsatile flow-induced deformation of the EG regulates blood flow in microvessels by altering its chemical properties via contraction of cortical actin web, activation of mechanosensitive ion channels and elevation of cytosolic calcium concentration with stimulation of eNOS.	Goligorsky (2022)
2021	Cells exposed to the unidirection shear stress exhibited a thicker EG and enhanced turnover of sialic acids which was reduced in cells cultured under oscillatory shear stress. Physiological unidirection shear stress, but not disturbed oscillatory shear stress, enhanced Nrf2 (nuclear factor erythroid 2-related factor 2)-mediated expression of antioxidant enzymes.	Psefteli et al. (2021)
2021	When exposed to fluid shear stress, fetoplacental endothelial cells have morphological changes, including elongating and re-orientating to the direction of flow. Fluid shear stress induces production of endothelial cell-derived vasoactive mediators, such as NO, VEGF and ATP.	Morley et al. (2021)
2021	Disturbed flow, characterized by low laminar shear stress or oscillatory shear stress, promotes endothelial activation by upregulating the expression of adhesion molecules, such as vascular cell adhesion molecule-1 (VCAM-1) and intercellular adhesion molecule-1 (ICAM-1), and the secretion of pro-inflammatory cytokines and chemokines. Cartilage oligomeric matrix protein (COMP) acted as a signaling molecule in the endothelium, amplifying the inflammatory response to the disturbed flow.	Lv et al. (2021)
2020	Increased arterial wall shear stress caused by a rapid flow change after a few minutes of ischemia initiates signal pathways that stimulate the change of the mechanical properties of the arterial wall. The artery then responds by increasing the diameter, hence initiating dilation and decreasing the flow speed along with the wall shear stress. Thus, arterial flow mediated dilation is an important indicator of vascular endothelial function.	Sidnawi et al. (2020)
2020	Shear stress alters the levels of molecules involved in embryonic development and angiogenesis. Low or oscillatory shear stress activation of a network drives angiogenesis and atherosclerosis, whereas high shear stress is inhibitory.	Souilhol et al. (2020)
2019	Disturbed shear stress induced by vortices leads to a sustained, stochastic NF-κB activation, which in turn promotes inflammatory responses and vascular remodeling. Moreover, NF-κB activity in low oscillatory shear stress and low pulsatile shear stress region is significantly higher than that in high pulsatile shear stress region.	Baeriswyl et al. (2019)
2019	The EG is redistributed when exposed to the flow shear stress and the changes in blood flow velocities may activate the signaling channels via the pertinent conformational changes of GAG chains.	Jiang et al. (2019a)
2019	Disturbed flow, generated from a microfluidic system with integrated ridge-shaped obstacles, changes the orientation angle of actin stress fibers and reduces the nuclear size while increases the nuclear circularity.	Tovar-Lopez et al. (2019)

**FIGURE 1:**
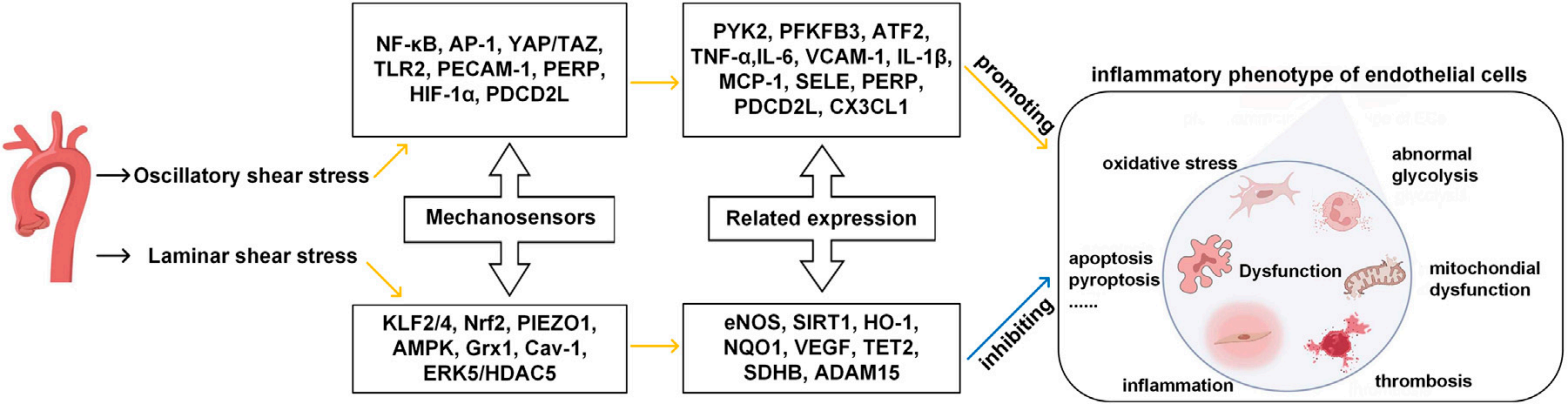
Effects of different patterns of shear stress on endothelial cell function in blood vessels. Yellow line indicates promotion, blue line indicates inhibition. (Modified from reference (Cheng et al., 2023) under a Creative Commons Attribution 4.0 International [CC BY 4.0) license].

The endothelial cells could sense changes upon shear stress and transit between quiescent and activated states (Collins and Tzima, 2011). As shown in Figure 1, mechanosensors first convert physical signals from different flow patterns into biochemical signals and then couple the signals with downstream signaling pathways, thereby inducing the expression of related genes and altering endothelial function and vascular remodeling.

Some of these mechanosensors can work as signal transduction molecules and transcription factors. For example, NF-κB (nuclear factor-κB), AP-1 (activator protein 1), YAP/TAZ (yes-associated protein/transcriptional co-activator with PDZ-binding motif), HIF-1α (hypoxia-inducible factor 1α), KLF2/4 (krüppel-like factor) and Nrf2 (nuclear factor-E2-related factor 2) can perceive extracellular signals such as oxygen levels and inflammatory cues, as they convert mechanical forces from the blood flow to cytoplasm. Specifically, when ECs are exposed to laminar shear stress, Nrf2 can protect ECs from oxidative stress by upregulating the expression of detoxifying enzymes, antioxidant enzymes, and proteins (Mann and Forman, 2015; Ishii et al., 2021). KLF2 is another important mediator of endothelial anti-inflammatory and anti-thrombotic properties (Boon and Horrevoets, 2009; Marin et al., 2013). In the OSS conditions, ECs can activate transcription factors (like NF-κB (Baeriswyl et al., 2019)), decrease smooth muscle cells contraction marker genes (such as smooth muscle *α*-actin and myocardin) and induce pro-inflammatory phenotype of the smooth muscle cells (such as the expressions of VCAM-1, IL-6, and MCP-1).

Some mechanosensors, including PIEZO1 (piezo-type mechanosensitive ion channel component 1), AMPK (AMP-activated protein kinase), TLR2 (toll-like receptors 2), Grx1 (glutenin 1), Cav-1 (caveolin-1), ERK5 (extracellular signal-regulated kinase), PECAM-1 (platelet/endothelial cell adhesion molecule-1), are signaling proteins, cell membrane proteins and enzymes which participate in intracellular signal transduction and regulation and maintain the stable structure and well-functioning of ECs. When ECs are exposed to laminar shear stress, the PIEZO1, a receptor ion channel anchored in the EC membrane, rapidly induces the activation of eNOS, thereby producing vasodilator NO through the upregulation and increased activity of eNOS, which has a profound impact on the proliferation and apoptosis of endothelial cells. When ECs are exposed to disturbed pulsatile flow, PECAM-1 senses the flow mechanical forces via the phosphorylation of two tyrosine residues of the cell-cell adhesion sites (Moriguchi and Sumpio, 2015). PECAM-1 can also interact with adhesion molecules such as ICAM-1 and VCAM-1 (Fu et al., 2023), and intensify the attachment of leukocyte to ECs. Molecular structures, signaling mechanisms, and the cellular environment can all influence the sensitivities of the mechanosensors in terms of signal perceiving accuracy and response speed to external stimuli. In the laminar shear stress environment, endothelial cells are anti-inflammatory, anti-oxidant and anti-thrombotic, the glycolysis process is improved, mitochondria are in homeostasis state, and the intracellular permeability is decreased. By contrast, in the oscillatory shear stress environment, endothelial cells are pro-inflammatory, pro-oxidative and pro-thrombotic, the glycolysis is abnormal, mitochondria are in dysfunction state, and the intracellular permeability is increased.

## 3 Interaction between EG and blood flow

**FIGURE 2:**
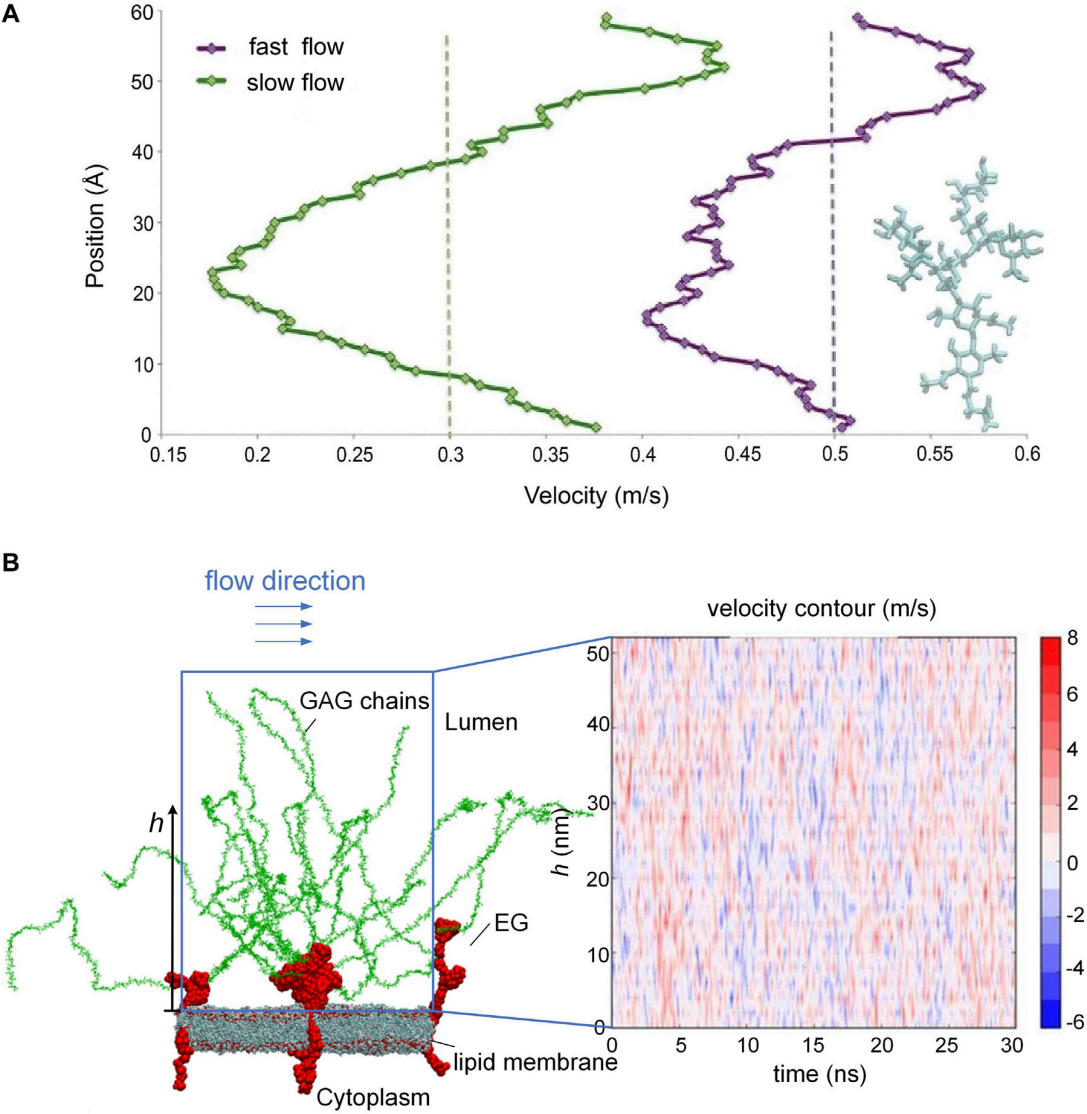
Blood flow velocity along the height of the GAG chains and oscillating velocity distributions. **(A)** Velocity drops as the shape of GAG chains becomes increasingly dendritic. **(B)** Alteration of blood flow velocity due to the conformational changes of the GAG chains. [Modified from references (Pikoula et al., 2018; Jiang et al, 2017) under a Creative Commons Attribution 4.0 International (CC BY 4.0) license].

The endothelial glycocalyx on the endothelial cell surface is the most inner layer of the endothelium and is in direct contact with circulating blood. Such a special location enables the EG to modulate blood flow by adjusting roughness via creating a layer of variable porosity and variable thickness. In an early *in vivo* study, the EG was demonstrated to exert extra resistance on the blood flow (Pries et al., 1997). To reveal the interactions between the EG and the blood flow, a series of large-scale molecular dynamics simulations *in vitro* were conducted to mimic the behavior of EG and the flow by tracking the trajectories of the atoms in the simulation system (Pikoula et al., 2018; Jiang et al., 2019a). By using such an atomic-scale method, the researchers were able to reproduce the microvascular events occurring on the endothelium, especially the flexible motions of GAG chains in response to the blood flow, which were difficult to observe by experimental methods. Scrutiny of velocity profile of the blood flow along the height of the GAG chains *in vitro* suggested that the blood velocity begins to drop particularly as the shape of the GAG chains becomes increasingly dendritic (Figure 2A) (Pikoula et al., 2018), and the loss or impairment of GAG chains could alter the surrounding blood flow field and fluid shear stress (Jiang et al., 2017; Jiang et al., 2021a). The alteration in the blood flow velocity profile occurs constantly (Figure 2B) as long as the conformational changes of the GAG chains by the flow happens, as reported in Ref. (Jiang et al., 2017). The passive modulation of the EG to the blood flow resistance maintains the blood homogeneity in the microvasculature. The shedding or loss of local EG could cause blood flow redistribution at the bifurcation (Machin et al., 2019), and the presence of heparinase results in glycocalyx degradation, thereby interfering with angiogenesis and perfusion (Ainslie et al., 2005).

The EG is a highly negatively charged brush-like structure with a thickness ranging from a few hundreds of nanometers to several micrometers (Squire et al., 2001). The distribution of endothelium could be modified or reorganized by the fluid shear stress via the EG. For example, Yao et al. (2007) reported that in cultured endothelial cells, heparan sulfate proteoglycans, a major component of the EG, redistribute from a uniform surface profile to a distinct peripheral pattern with most molecules detected above cell-cell junctions after 24-h exposure to the flow. The redistribution of the EG reduces the shear gradients that the cell surface experiences, helping the endothelial cells adapt to the forces from the blood flow. The strength of fluid shear stress also influences the synthesis of GAGs. For example, low laminar shear stress has been repeatedly shown to impair EG and in particular to lead to hyaluronic acid degradation in the EG (Koo et al., 2013). Giantsos-Adams et al. (2013) proved that shear stress enhanced heparan sulfate synthesis compared to static controls after enzymatic removal of heparan sulfate. A study with cultured endothelial cells by Koo et al. (2013) detected increased heparan sulfate expression and even spatial distribution on the apical surface under relatively high shear conditions without shear reversal but reduced heparan sulfate expression and irregular cell distribution under low shear conditions with shear reversal. It is noteworthy that the EG *in vitro* is thinner than its *in vivo* counterpart, as cultured endothelial cells are conventionally subjected to dehydration that would lead to glycocalyx collapse (Ebong et al., 2011).

## 4 Endothelium damage and restoration

**FIGURE 3:**
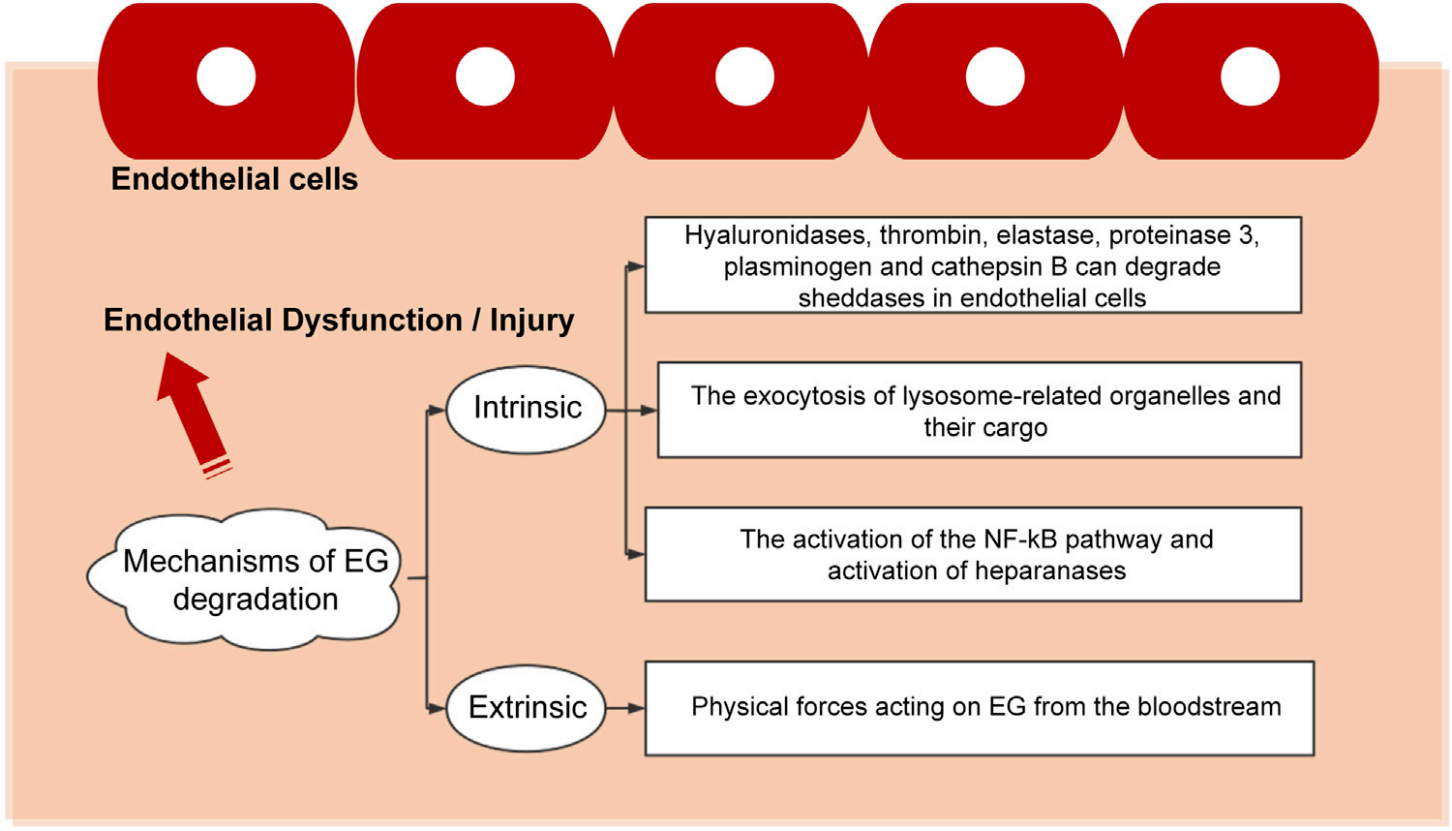
Pathways leading to the damages of endothelial cells.

The pathogenesis of vascular-related diseases is multifactorial, with most of them related to EG and influenced by integrity and functional expression the EG. EG is a fragile structure that can be damaged by various stimuli, leading to endothelial dysfunction characterized by enhanced vascular permeability, impaired vasodilation, increased leucocyte-endothelium interactions, thrombosis, and vascular inflammation. The existing results indicate that the mechanisms of EG degradation can be divided into two large categories, i.e., intrinsic and extrinsic, as detailed in Figure 3.

The interaction between the endothelial cells and the microenvironment determines the physiological status of cells. The degradation or other type of destruction mode causes the dysfunction or death of the endothelial cells and leads to a series of diseases. Disruption to the EG was observed under various pathological conditions, including infectious diseases (such as sepsis, dengue virus, flaviviruses, human corona viruses and malaria), cardiovascular diseases (such as atherosclerosis, stroke, chronic venous disease, hypertension and aging), diabetes, trauma and cancer (Weinbaum et al., 2021). The commonly used method to determine whether EG is damaged is to detect the concentrations of EG fragments, such as hyaluronic acid, heparan sulfate and syndecan-1, in the plasma. Such fragments are the biomarkers for EG shedding. For instance, in patients with sepsis caused by pneumonia, the concentration of plasma syndecan-1 increases over time, and the syndecan-1 is the only EG biomarker that is moderately correlated with inflammation (Smart et al., 2018). In rats, the circulating levels of syndecan-1 and hyaluronic acid are significantly reduced in the antithrombin-treated group (Iba et al., 2018).

In acute inflammation, the entire EG unit, including the core protein and the GAG chains, is completely destroyed, whereas, in chronic inflammation, the core protein is retained but the associated GAG chains are injured. Restoration of damaged EG and protection against further damage are promising targets for the treatment of chronic vascular diseases (Becker et al., 2010). As such, two therapeutic strategies can be formed to maintain the integrity of the EG: one is to prevent glycocalyx degradation (Zullo et al., 2016), and the other is to promote the glycocalyx biosynthesis (Henry and Duling, 1999; Coccheri and Mannello, 2013). To implement these therapies, agents with special functions can be used. For example, protective agents, such as heparin, heparanase antagonists, antithrombins and corticosteroids, can be used to fight inflammation and thereby prevent glycocalyx shedding (Rubio-Gayosso et al., 2006; Chappell et al., 2009; Brands et al., 2010). However, the clinical efficacy of using agents to prevent EG shedding remains unproven and may even be harmful, as the doses of the anti-inflammatory agents are hard to control and the anti-inflammatory agents may also cause unforeseen problems. Alternatively, as mentioned previously, endothelial cells behave differently under different blood flow patterns, which leaves the possibility to repair or maintain the endothelial cell functionality by applying flows with different flow characteristics. As demonstrated in previous *in vitro* experiments, when vascular smooth muscle cells and endothelial cells are co-cultured, flows with laminar shear stress can inhibit the dedifferentiation, migration and proliferation of vascular smooth muscle cells while maintaining the contractile phenotype (Hastings et al., 2007; Tsai et al., 2009; Sakamoto et al., 2011).

## 5 Discussion

Blood flow and the resulting fluid shear stress play a critical role in maintaining endothelial functionality. Endothelial cells covered by the EG are an important medium to convey and convert flow signals. In this review, we summarized the latest findings about the interactions between the blood flow and the endothelium, with special focus given to the modified flow field by EG configurational changes. The shear stress modulates the functionality of endothelial cells, from the activation of mechanosensors to the cellular phenotypes of vascular endothelial cells. To treat chronic vascular diseases, therapies focusing on restoring EG or preventing EG from further damage can be considered. The restoration and prevention could be realized via applying appropriate fluid shear stress to the endothelial cells. Mechanosensors like PECAM-1 and mechanosensitive ion channels, such as PIEZO1, can be targeted to modulate endothelial cell responses to flow (Ridone et al., 2019). Thus, drugs or therapies that could control the activities of these mechanosensors may become novel treatment options. Intervention of flow and shear stress patterns *in vivo* or in human bodies is no doubt a promising strategy to treat vascular diseases but also a challenging topic deserving significant additional effort.
